# Supplementary figures and images for: The allelic rice immune receptor Pikh confers extended resistance to strains of the blast fungus through a single polymorphism in the effector binding interface
Source: PLoS Pathog. 2021 Mar 1;17(3):e1009368. doi: 10.1371/journal.ppat.1009368 (PMC7951977; doi:10.1371/journal.ppat.1009368)

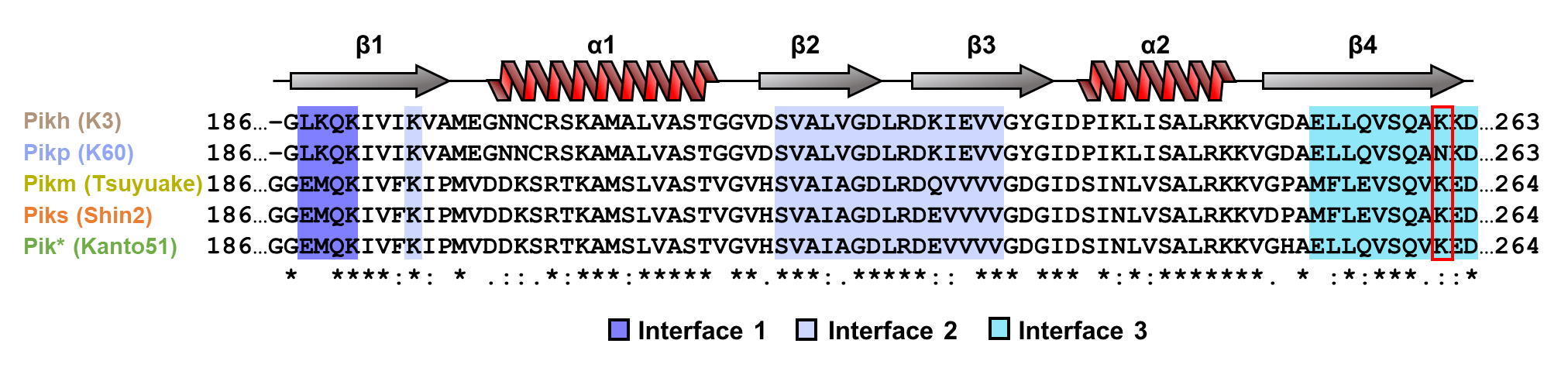

Supplement: S1 Fig — Amino acid sequence alignment of Pikh-1, Pikp-1, Pikm-1, Piks-1 and Pik*-1. Secondary structure features of the HMA fold are shown above, and the residues located to the binding interfaces as described in [20] are highlighted. The Pikh-HMA polymorphic position (residue 261), located in binding interface three, is indicated in a red square. (TIF) [file ppat.1009368.s001.tif]

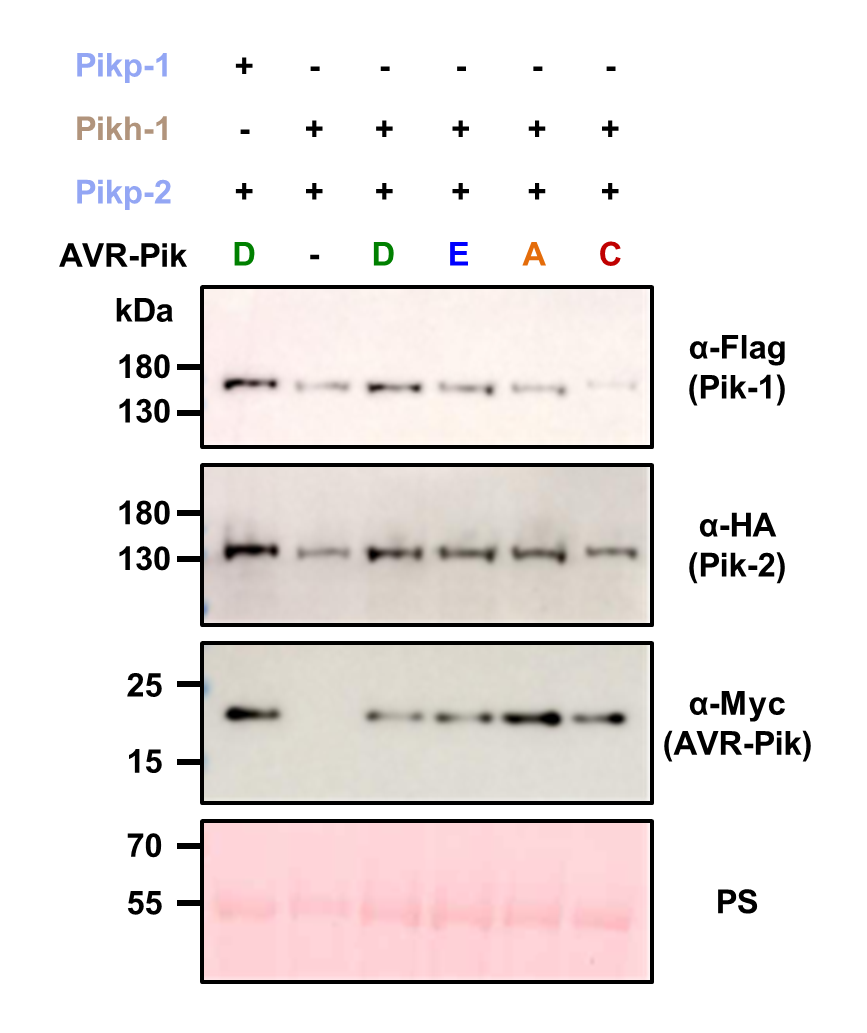

Supplement: S2 Fig — Plant lysate was probed for the expression of Pikh-1, Pikp-2 (100% identical to Pikh-2) and AVR-Pik effectors using anti-FLAG, anti-HA and anti-Myc antisera, respectively. Accumulation of the control Pikp-1/Pikp-2/AVR-PikD proteins were also measured as a comparison. Total protein extracts were visualized by Ponceau Staining (PS). (TIF) [file ppat.1009368.s002.tif]

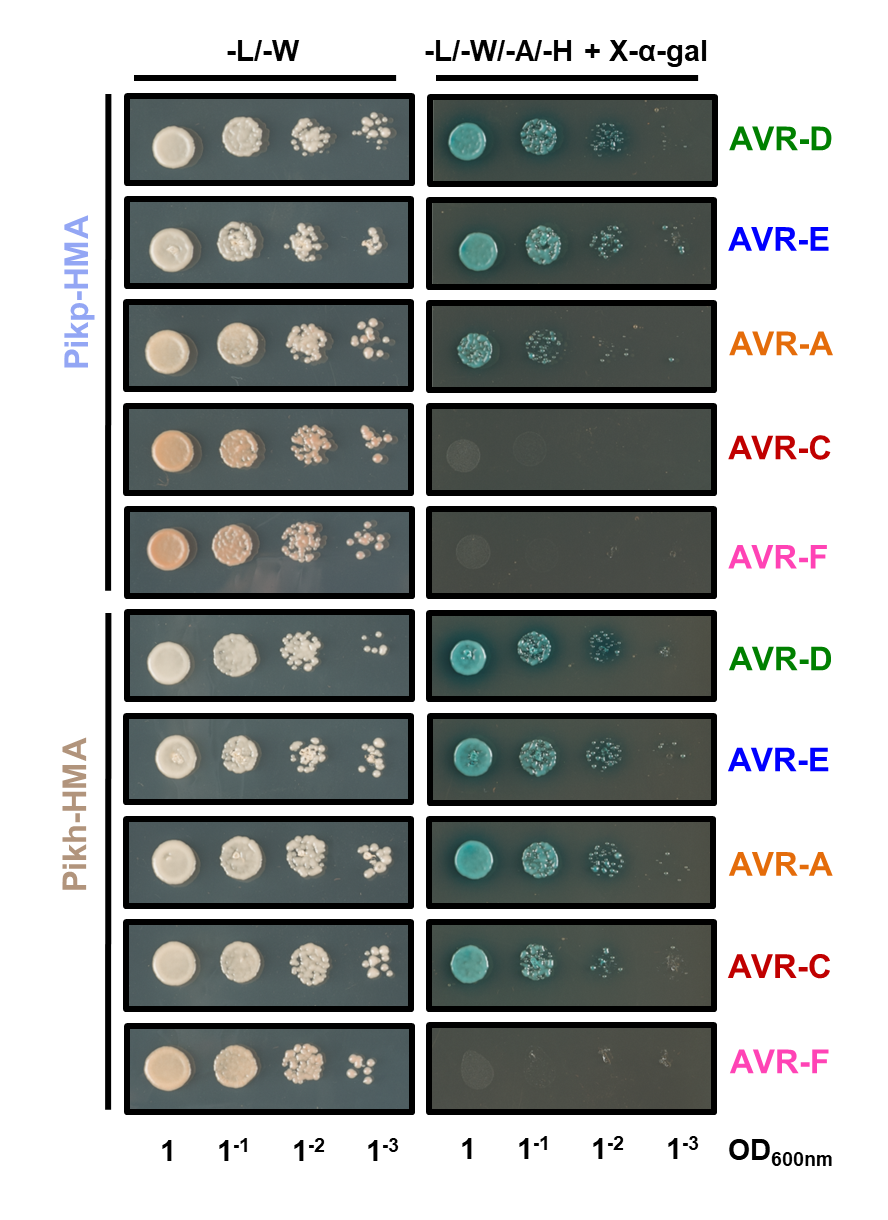

Supplement: S3 Fig — For each combination of HMA/AVR-Pik, 5μl of yeast were spotted and incubated for ~84 h in double dropout plate for yeast growth control (left) and quadruple dropout media supplemented with X-α-gal (right). Growth, and development of blue colouration, in the selection plate are both indicative of protein:protein interaction. HMA domains were fused to the GAL4 DNA binding domain, and AVR-Pik alleles to the GAL4 activator domain. Each experiment was repeated a minimum of three times, with similar results. (TIF) [file ppat.1009368.s003.tif]

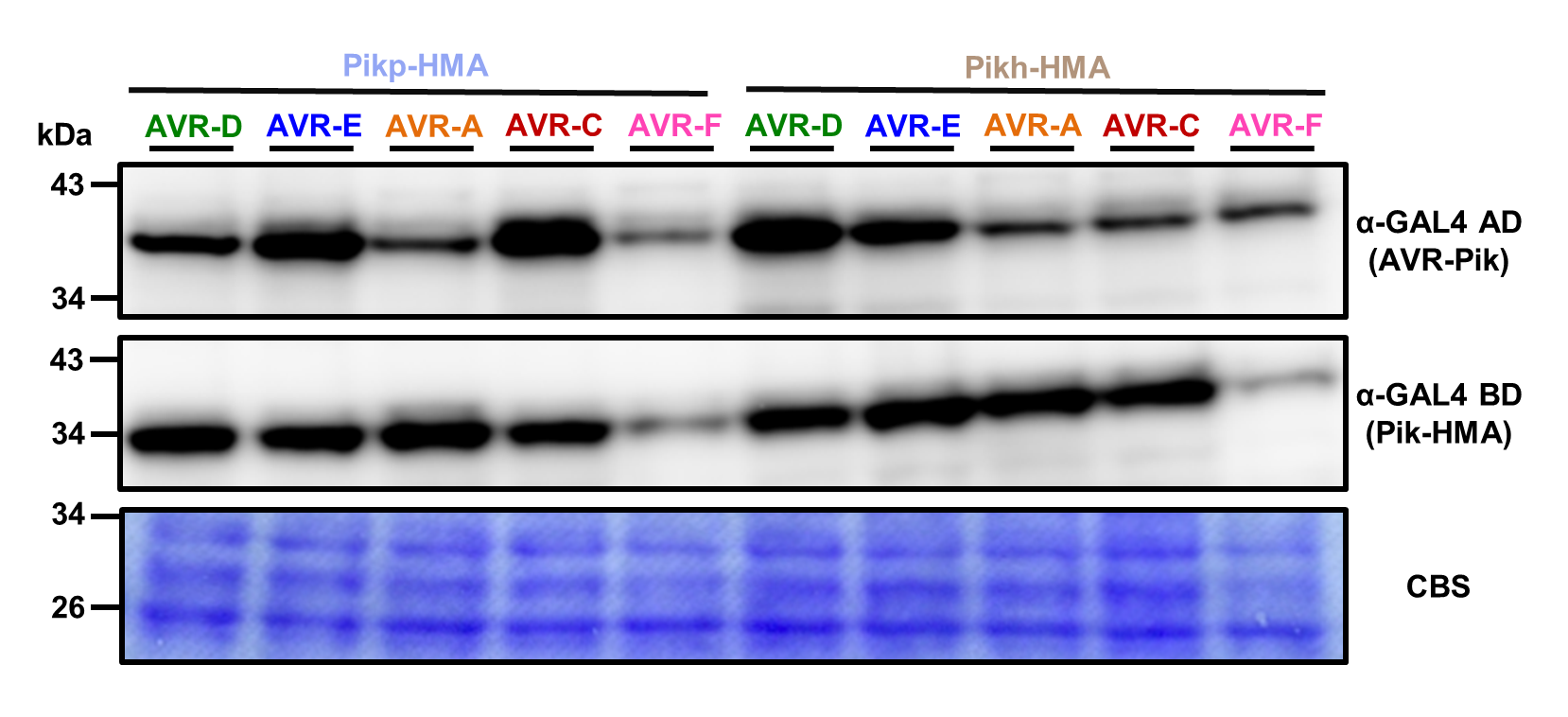

Supplement: S4 Fig — Yeast lysate was probed for the expression of AVR-Pik effectors and HMA domains using anti-GAL4 activation domain (AD) and anti-GAL4 DNA binding domain (BD) antibodies, respectively. Total protein extracts were coloured with Coomassie Blue Stain (CBS). (TIF) [file ppat.1009368.s004.tif]

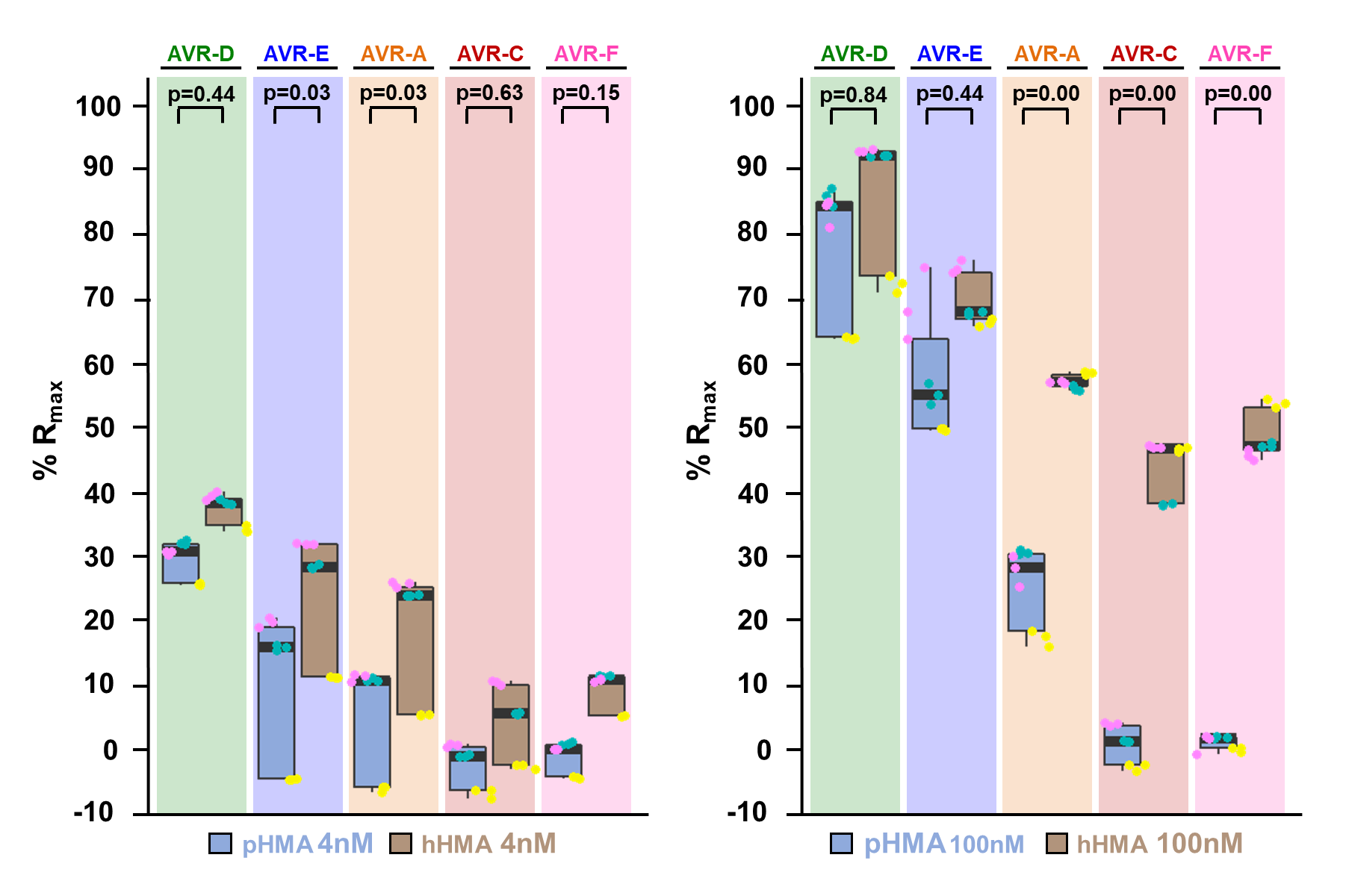

Supplement: S5 Fig — Measurement of Pikp-HMA and Pikh-HMA binding to AVR-Pik variants measured by surface plasmon resonance. The binding is expressed as %Rmax at HMA concentration of 4 nM (left) and 100 nM (right). Pikp-HMA and Pikh-HMA are represented by blue and brown boxes, respectively. For each experiment, three biological replicates with three internal repeats were performed and the data are presented as box plots. The centre line represents the median, the box limits are the upper and lower quartiles, the whiskers extend to the largest value within Q1-1.5× the interquartile range (IQR) and the smallest value within Q3 + 1.5× IQR. All the data points are represented as dots with distinct colours for each biological replicate. (TIF) [file ppat.1009368.s005.tif]

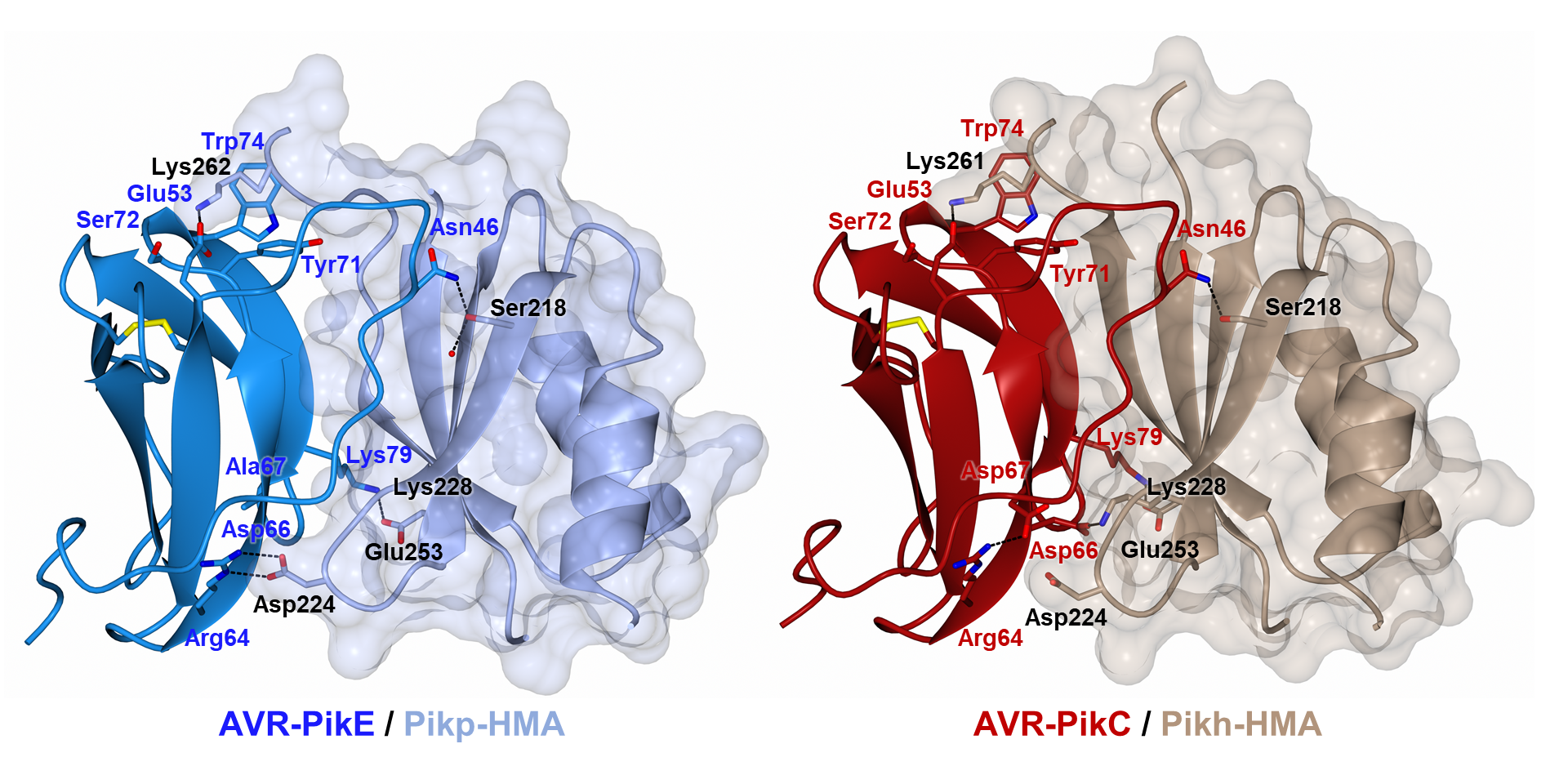

Supplement: S6 Fig — Schematic representation of the structure of Pikh-HMA in complex with AVR-PikC (right). The structure of Pikp-HMA bound to AVR-PikE (PDB: 6G11) from [20] is included for comparison (left). HMA domains are presented as cartoon ribbons with selected side chains as cylinders; the molecular surface of the HMA domain is also shown. Pikh-HMA and Pikp-HMA are coloured in brown and ice blue, respectively. The effectors are shown in cartoon ribbon representation, with selected side chains as cylinders. AVR-PikC and AVR-PikE are coloured in crimson and bright blue, respectively. Hydrogen bonds/salt bridges are shown as black dashed lines and disulfide bonds as yellow cylinders. For clarity, of the two molecules of Pik-HMA present in the complex, only the one making extensive contacts with the effector is shown. (TIF) [file ppat.1009368.s006.tif]

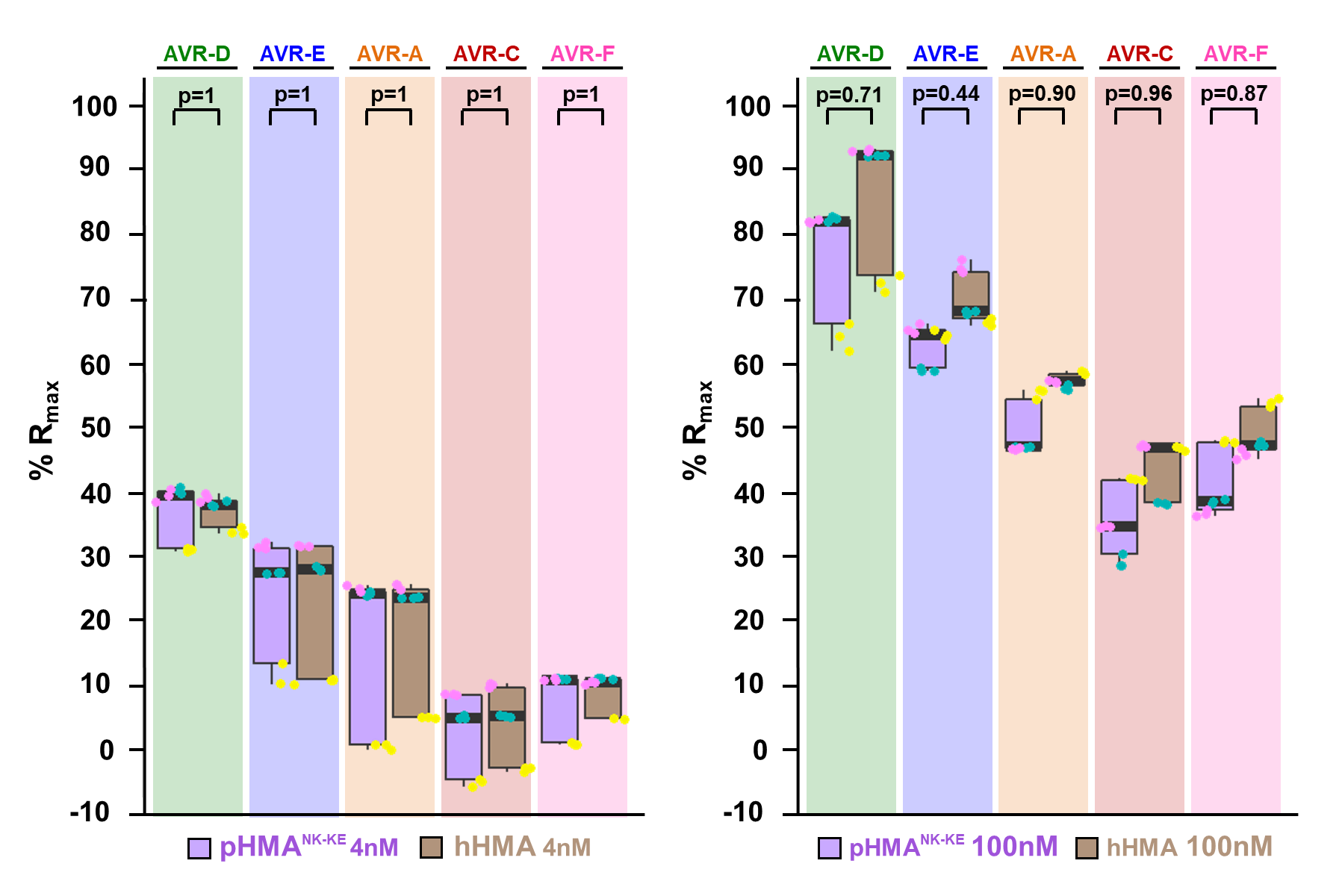

Supplement: S7 Fig — Measurement of Pikp-HMANK-KE and Pikh-HMA binding to AVR-Pik variants measured by surface plasmon resonance. The binding is expressed as %Rmax at HMA concentration of 4 nM (left) and 100 nM (right). Pikp-HMANK-KE and Pikh-HMA are represented by purple and brown boxes, respectively. For each experiment, three biological replicates with three internal repeats were performed and the data are presented as box plots. The centre line represents the median, the box limits are the upper and lower quartiles, the whiskers extend to the largest value within Q1-1.5× the interquartile range (IQR) and the smallest value within Q3 + 1.5× IQR. All the data points are represented as dots with distinct colours for each biological replicate. Data for Pikh-HMA is also presented in S5 Fig and were collected side-by-side at the same time. (TIF) [file ppat.1009368.s007.tif]

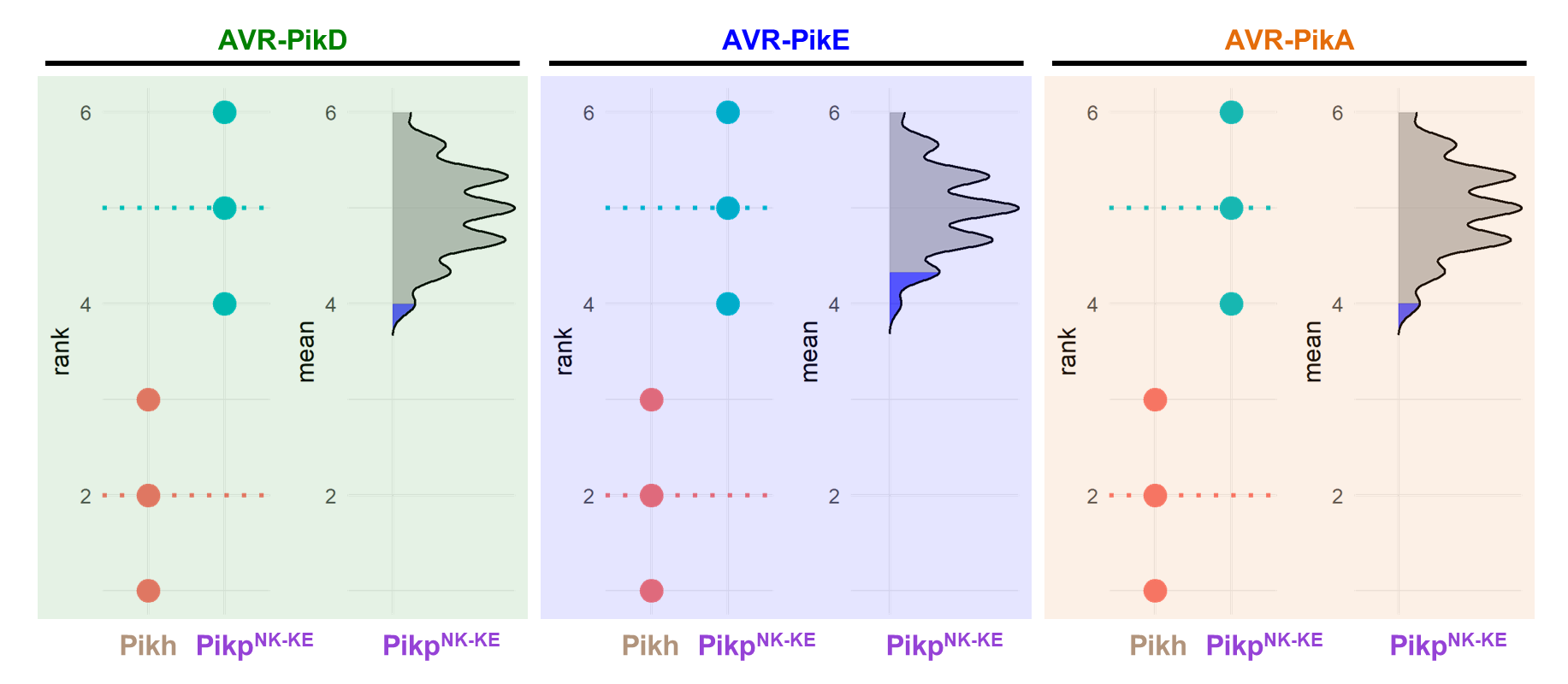

Supplement: S8 Fig — Statistical analysis by estimation methods of the cell-death assay for Pikh and PikpNK-KE. For each effector, the panel on the left represents the ranked data (dots) for each NLR, and their corresponding mean (dotted line). The size of the dots is proportional to the number of observations with that specific value. The panel on the right shows the distribution of 1000 bootstrap sample rank means for PikpNK-KE. The blue areas represent the 0.025 and 0.975 percentiles of the distribution. The responses of Pikh and PikpNK-KE are considered significantly different if the Pikh rank mean (dotted line, left panel) falls beyond the blue regions of the PikpNK-KE mean distribution. (TIF) [file ppat.1009368.s008.tif]

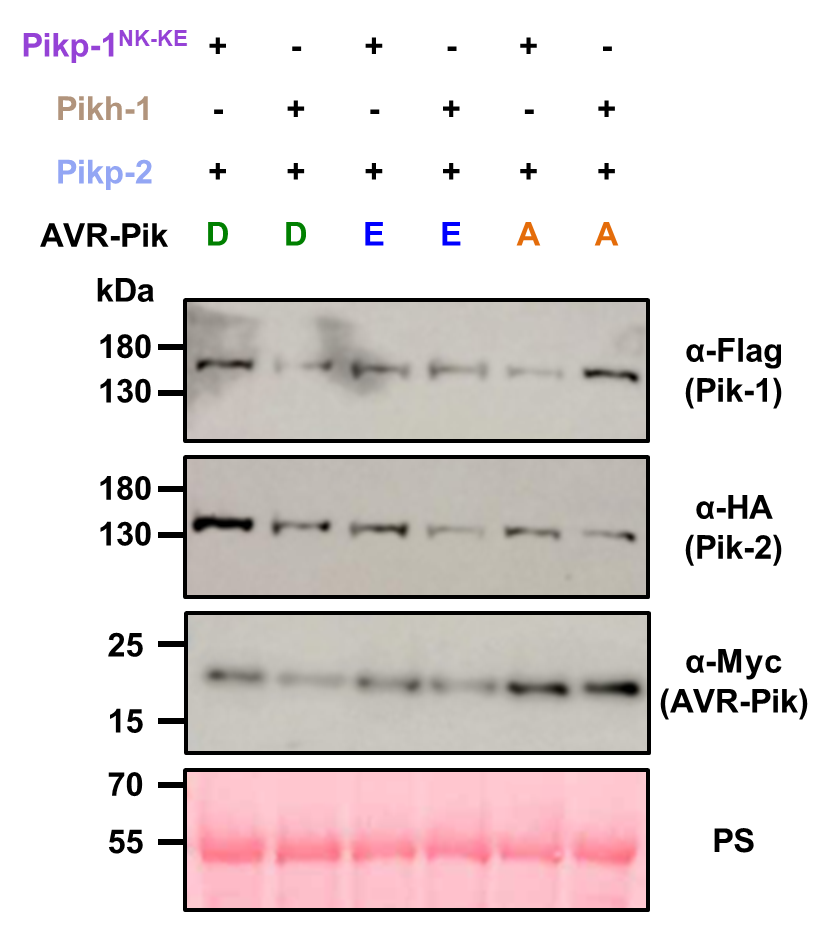

Supplement: S9 Fig — Plant lysate was probed for the expression of PikpNK-KE-1/Pikh-1, Pikp-2 and AVR-Pik effectors using anti-FLAG, anti-HA and anti-Myc antiserum, respectively. Total protein extracts were visualised by Ponceau Staining (PS). (TIF) [file ppat.1009368.s009.tif]
