# Supplementary material for: The allelic rice immune receptor Pikh confers extended resistance to strains of the blast fungus through a single polymorphism in the effector binding interface
Source: PLoS Pathog. 2021 Mar 1;17(3):e1009368. doi: 10.1371/journal.ppat.1009368 (PMC7951977; doi:10.1371/journal.ppat.1009368)
Supplement: S1 Table — (DOCX) [file ppat.1009368.s010.docx]

| **Data collection statistics** | |
| --- | --- |
| Wavelength (Å) | 0.98 |
| Space group | *P* 2_1_ 2_1_ 2_1_ |
| Cell dimensions *a*, *b*, *c* (Å) | 65.35, 83.12, 107.84 |
| Resolution (Å)* | 107.84-2.30 (2.38-2.30) |
| R_merge_  (%) | 6.1 (92.7) |
| *I* /σ*I* | 22.7 (2.5) |
| Completeness (%) | 96.4 (100.0) |
| Unique reflections | 25847 (2570) |
| Redundancy | 13.1 (12.0) |
| CC(1/2) (%) | 100.0 (86.4) |
|  |  |
| **Refinement and model statistics** | |
| Resolution (Å) | 65.92-2.30 (2.36-2.30) |
| R_work_/R_free_ (%) | 21.0/25.5 (26.6/31.1) |
| No. atoms |  |
| Protein | 3464 |
| Water | 116 |
| B-factors |  |
| Protein | 61.5 |
| Water | 55.2 |
| R.m.s deviations |  |
| Bond lengths (Å) | 0.008 |
| Bond angles (^°^) | 1.486 |
| Ramachandran plot (%)** |  |
| Favoured | 97.93 |
| Allowed | 2.07 |
| Outliers | 0 |
| MolProbity Score | 1.52 (99^th^ percentile) |
|  |  |
| * The highest resolution shell is shown in parenthesis.  ** As calculated by MolProbity | |
