# Supplementary material for: The allelic rice immune receptor Pikh confers extended resistance to strains of the blast fungus through a single polymorphism in the effector binding interface
Source: PLoS Pathog. 2021 Mar 1;17(3):e1009368. doi: 10.1371/journal.ppat.1009368 (PMC7951977; doi:10.1371/journal.ppat.1009368)
Supplement: S2 Table — For Pikh-HMA/AVR-PikC, chains E and F were used. (DOCX) [file ppat.1009368.s011.docx]

| **Complex (compared to Pikh/AVR-PikC)** | **AVR-Pik** | **HMA** | **Overall** |
| --- | --- | --- | --- |
|  | r.m.s.d. - Å  (no. of residues) | r.m.s.d. - Å  (no. of residues) | r.m.s.d. - Å  (no. of residues) |
| Pikp/AVR-PikE | 0.31 (82 aa) | 0.61 (72 aa) | 0.70 (154 aa) |
| Pikm/AVR-PikE | 0.46 (81 aa) | 0.92 (74 aa) | 0.92 (165 aa) |
